# Tuberculous Lymphadenitis in Northern Ethiopia: In a Public Health and Microbiological Perspectives

**DOI:** 10.1371/journal.pone.0081918

**Published:** 2013-12-09

**Authors:** Fantahun Biadglegne, Weghata Tesfaye, Ulrich Sack, Arne C. Rodloff

**Affiliations:** 1 College of Medicine and Health Sciences, Bahir Dar University, Bahir Dar, Ethiopia; 2 Institute of Medical Microbiology and Epidemiology of Infectious Diseases, University Hospital, University of Leipzig, Leipzig, Germany; 3 Institute of Clinical Immunology, University Hospital, University of Leipzig, Leipzig, Germany; 4 College of Medicine and Health Sciences, University of Gondar, Gondar, Ethiopia; 5 Translational Centre for Regenerative Medicine (TRM)-Leipzig, University of Leipzig, Leipzig, Germany; National Institute of Medical Research, United Kingdom

## Abstract

**Background:**

The actual burden and causative agent of tuberculous lymphadenitis (TBLN) cases is not well known due to lack of strong surveillance system and diagnostic facilities in Ethiopia. This study was conducted to determine the prevalence of TBLN, its causative agent and risk factors for acquiring this infection.

**Methods:**

A cross-sectional study was conducted from April to May 2012 at four main hospitals and one diagnostic clinic located in northern Ethiopia. Fine needle aspirates (FNAs) from TBLN suspects were taken for acid fast bacilli (AFB) microscopy, culture and molecular typing.

**Results:**

Among 437 aspirates, culture yielded AFB in 226 (51.7%) of cases. Sixty one culture negative cases (30.5% of 200 cases) were positive by Xpert MTB/RIF test. Moreover, a rifampicin resistant AFB was detected from culture negative cases. The overall prevalence of FNAs positive TBLN cases was 65.8 %. The BacT/AlerT 3D system proved to be a more rapid method with higher recovery rate than Lowenstein-Jensen (L-J) and/or Gottsacker media (P<0.0001). Molecular typing identified all culture positive isolates as *M.tuberculosis*. The main risk factors for TBLN were pediatric age (OR 2.8, 95% CI, 1.09- 7.05) and cough (OR 2, 95%CI, 1.09-3.7).

**Conclusions:**

The results of this study revealed a high prevalence of TBLN in the study sites and that pediatric age and cough are key predictors of the disease. TBLN is an important public health problem that needs to be addressed in the area. It is important to note that MDR strains of TB could be involved and aetiological confirmation and drug sensitivity testing of TBLN isolates should be expanded. Further studies on the *M.tuberculosis* lineages, circulating strains and transmission dynamics, are recommended.

## Introduction

The incidence of extra-pulmonary tuberculosis (EPTB) among patients with newly diagnosed tuberculosis (TB) has been increasing in Ethiopia, with a majority of patients being diagnosed with TBLN [1].Ethiopia ranks third in terms of EPTB patients worldwide, with TBLN being the most common presentation [2]. The reasons for the increased frequency of TBLN in Ethiopia are unknown. The term EPTB has been used to describe isolated occurrences of TB at body sites other than the lungs [3], which includes the lymph nodes, pleura, bones, joints, brain, meninges, gastrointestinal organs, liver; genitourinary organs, peritoneum and pericardium. The lymph nodes commonly affected by TBLN include cervical, axillar, inguinal and abdominal sites. Cervical lymph nodes are the most commonly affected group of nodes reported [4].Among members of the *Mycobacterium tuberculosis* complex (MTBC), *M.tuberculosis* and *M.bovis* have been reported as the most frequent causative agents in TBLN [5-8]. As a result, identification of species causing TBLN is an important step towards establishing control measures based on the most common causative agent.

Diagnosis of TBLN is not easy, especially when clinical feature is indicative but bacteriological evidence is missing. Laboratory diagnosis of TBLN remains mainly in a stage of fine needle aspiration cytology (FNAC) and Ziehl Nielsen (ZN) smear in Ethiopia. However, ZN smear and FNAC lacks sensitivity and specificity respectively [9,10]. Moreover, these methods are limited due to lack of species identification and drug susceptibility testing. Culture for *Mycobacterium* species is not available as a routine test in Ethiopia, not even for cases with suspected infection with drug resistant strains. Consequently, patients with granulomatous lymph adenopathy of unknown origin will either have specific therapy delay or will wrongly take anti-TB treatment.

Studies conducted using FNAC confirmed that TBLN is endemic in Ethiopia with prevalence rates varying from 56.8%-72.4% [11-14].However, these results should be interpreted with caution, where there are limited laboratory facilities for the definitive diagnosis of *Mycobacterium* species in the country. The current prevalence of TBLN and its associated risk factors as well as circulating species has not been clearly investigated in northern Ethiopia. With this background, the aims of this study were to determine the prevalence of TBLN, its causative agent and risk factors for acquiring this infection. 

## Materials and Methods

### Study design and subjects

This cross-sectional prospective study was conducted at four main hospitals (Felege Hiwot, Gamby, Gondar and Dessie) and at Bikat Diagnostic Clinic located in northern Ethiopia between April and May 2012. Patients with enlarged lymph nodes who were not responding to a two-week course of broad spectrum antibiotics and who were clinically and cytologically diagnosed as isolated TBLN (N=437) were included in this study. Pyogenic abscesses were excluded based on the clinical and cytomorphological features. A structured and pretested questionnaire was used to collect demographic characteristics of study subjects. The study was conducted with the consent of the patients after obtaining an institutional ethical clearance from research and publication committee ethical review board of the Bahir Dar University, Bahir Dar, Ethiopia. 

### Specimen collection, storage and transport

The fine needle aspirated samples were collected from lymph nodes of all patients. Fine needle aspirates (FNAs) were divided into two halves one for cytology and the other for culture. The cytological criteria for diagnosis of TBLN have been clearly defined [15]. The culture FNAs was placed into 1ml of phosphate buffer saline (PH=6.8) and stored at -80°C until transported to the laboratory in Leipzig, Germany. 

The specimens were packed on ice and transported according to regulation provided by the International Air Transport Association (http://www.iata.org/ads/issa/htm). 

### Processing and culture of aspirates

FNAs were processed for culture from May to July, 2012 according to the Deutsches Institut fur Normung (DIN) recommendations for the detection of mycobacteria by culture methods [16] in the Institute of Medical Microbiology and Infectious Disease, University of Leipzig, Germany. FNAs were liquefied by adding 10 ml of distilled water, then digested and decontaminated by using equal volumes (10ml) of N-acetyl-L cysteine (NALC)-NaOH solution (4% NaOH, 2.9% sodium citrate, and 0.5% NALC) for 20 minutes at room temperature on a shaker. After decontamination, aspirates were neutralized with phosphate buffer saline (pH 6.8) and concentrated by centrifugation at 3,300x*g* for 20 minutes. The concentrated aspirates were resuspended in 1ml of phosphate buffer saline and 0.5 ml of the aspirates was used for inoculation in to BacT/AlerT MP bottles. The bottles were supplemented with 0.5 ml of antibiotics based on the information provided by the manufacturer (bioMérieux SA, France). After inoculation, the bottles were incubated at 37°C for 12 weeks in an automated BacT/AlerT 3D incubator. Detection of Mycobacterium growth in the BacT/AlerT 3D system was based on the colorimetric detection of CO_2_, and the cultures were continuously monitored by the automated system. Each of the Lowenstein-Jensen (L-J) and Gottsacker slants were inoculated with 0.2 ml sediment and incubated at 37°C for 12 weeks. The L-J and Gottsacker tubes were read twice during the first week and followed by weekly basis until the 12^th^ week. Smears of the concentrated FNAs were prepared and stained using an auramine-rhodamine stain for fluorescent microscopy examination. The results were recorded according to the standard grading scale [17].The remaining FNAs concentrates were stored at -80°C for further investigation. Samples that were culture negative were examined by Xpert MTB/RIF assay (Cepheid, Sunnyvale, CA) for *M. tuberculosis* complex DNA and its susceptibility to rifampicin.

### Species Identification

#### DNA extraction

Isolates with the typical morphology of members of the *M. tuberculosis* complex were identified using DNA hybridization technology with a nitrocellulose strip assay for GenoType MTBC assay (Hain Life Science GmbH, Nehren, Germany) according to the manufacturer’s recommendations. For extraction of DNA, 1ml of liquid culture from the bottles were transferred in to eppendorf tubes, and then subjected to a heating block at 95°C for 20 minutes followed by sonication in an ultrasonic water bath for 15 minutes and then centrifugation. From the suspension 5 µl was directly used for PCR amplification.

#### GenoType MTBC assay

The amplification of DNA for genotyping was performed as recommended by the manufacturer. Briefly, for amplification 35μl of a primer nucleotide mixture, amplification buffer containing 5μl mMMgCl_2,_ 2.5μl sterile water, 2.5μl (1 unit) Taq DNA polymerase (ROCHE, Mannheim, Germany), and 5 μl of DNA in a final volume of 50μl were used. The amplification protocol consisted of 15 min at 95°C for denaturation, followed by 10 cycles comprising 30s at 95 °C and 2min at 58°C,an extra 20cycles comprising 25s at 95°C,40s at 53°C,and 40s at 70°C and finally it was extended for 8 min at 70°C . Hybridization of the single stranded, biotin-labeled amplicons to membrane-bound probes on the strip followed by addition of conjugate, and substrate to detect visible band patterns on the strip was performed manually using a shaking water bath, Memmert-SV1422 (Memmert GmbH & CO.KG, Schwabach, Germany) at 45°C. Then the strips were allowed to dry and interpreted according to the manufacturer’s recommendation for species identification. 

### Data management and analysis

All data were entered, cleared, and analyzed using the SPSS statistical software package, Version 16 (SPSS Inc., Chicago, IL, USA). This study used a linear regression model to analyze predictors of a successful culture. The McNemar modification of the chi-square test was used to assess differences in recovery of *M. tuberculosis* among different culture methods. Descriptive data analysis was used to visualize differences within the data and multivariate logistic regression was done to assess risk factors associated with TBLN in terms of the odds ratio and its 95% confidence interval (CI). Differences were considered significant when p-value is less than or equal to 0.05.

## Results

A total of 437 TBLN suspects were enrolled in this study. Female constituted 57.5% of the total study population.Their age ranged from 1 to 70 years, with a mean age of 28 years. The cervical lymph nodes were the most superficial lymph node group affected. The most prevalent clinical feature was matted in 76.5% (n=173) cases, with a majority of (129/173) being in the cervical region, followed by axillary (26/173). Fifty (22.1%) patients exhibited discrete adenopathy, in which 18.1% (n=41) were found to be in the cervical region (Table 1). 

**Table 1:** Lymph node regions of TBLN patients (n= 226), by sex, age, residence and clinical feature northern Ethiopia.

			Infected sites		
Variables	Cervical N (%)	Axilla N (%)	Inguinal N (%)	Other N (%)	Total N (%)
**Sex**					
**Male**	71(31.4)	16(7.1)	5(2.2)	4(1.8)	96(42.5)
**Female**	100(44.3)	17(7.5)	10(4.4)	3(1.3)	130(57.5)
**Age group in years**					
**0-14**	20(8.9)	4(1.8)	1(0.4)	1(0.4)	26(11.5)
**15-24**	48(21.2)	10(4.4)	5(2.2)	1(0.4)	64(28.2)
**25-34**	62(27.4)	14(6.2)	4(1.8)	0(0)	80(35.4)
**35-44**	21(9.4)	1(0.4)	3(1.3)	2(0.9)	27(12.0)
**45-54**	11(4.9)	1(0.4)	0(0)	2(0.9)	14(6.2)
**55-64**	7(3.2)	2(0.9)	1(0.4)	0(0)	10(4.4)
**>64**	2(0.9)	1(0.4)	1(0.4)	1(0.4)	5(2.2)
**Patients residence**					
**Urban**	52(23.0)	8(3.5)	4(1.8)	2(0.9)	66(29.2)
**Rural**	119(52.7)	24(10.6)	12(5.3)	5(2.2)	160(70.8)
**Clinical features**					
**Matted**	129(57.0)	26(11.5)	12(5.3)	6(2.7)	173(76.5)
**Discrete**	41(18.1)	6(2.7)	2(0.9)	1(0.4)	50(22.1)
**Other**	1(0.4)	1(0.4)	1(0.4)	0(0)	3(1.3)

N, number

Among all aspirates, 3 % (n=13) TBLN cases were AFB smear positive. Out of these, 2.5% (n=11) were culture positive. In this study, AFB smear positivity was more predictive of a positive culture result (P< 0.0001) in all culture methods. On the bases of culture, 51.7% (n=226) were positive but 45.8 % (n=200) failed to grow in all culture methods. Eleven (2.5%) FNAs were contaminated with gram positive bacteria. Sixty one culture negative cases were positive by Xpert MTB/RIF test. Of this 1 was a rifampicin resistant AFB. The overall prevalence of lymph node aspirates positive (culture and/or Xpert MTB/RIF test) TBLN cases were 65.7 %. 

**Figure 1:**
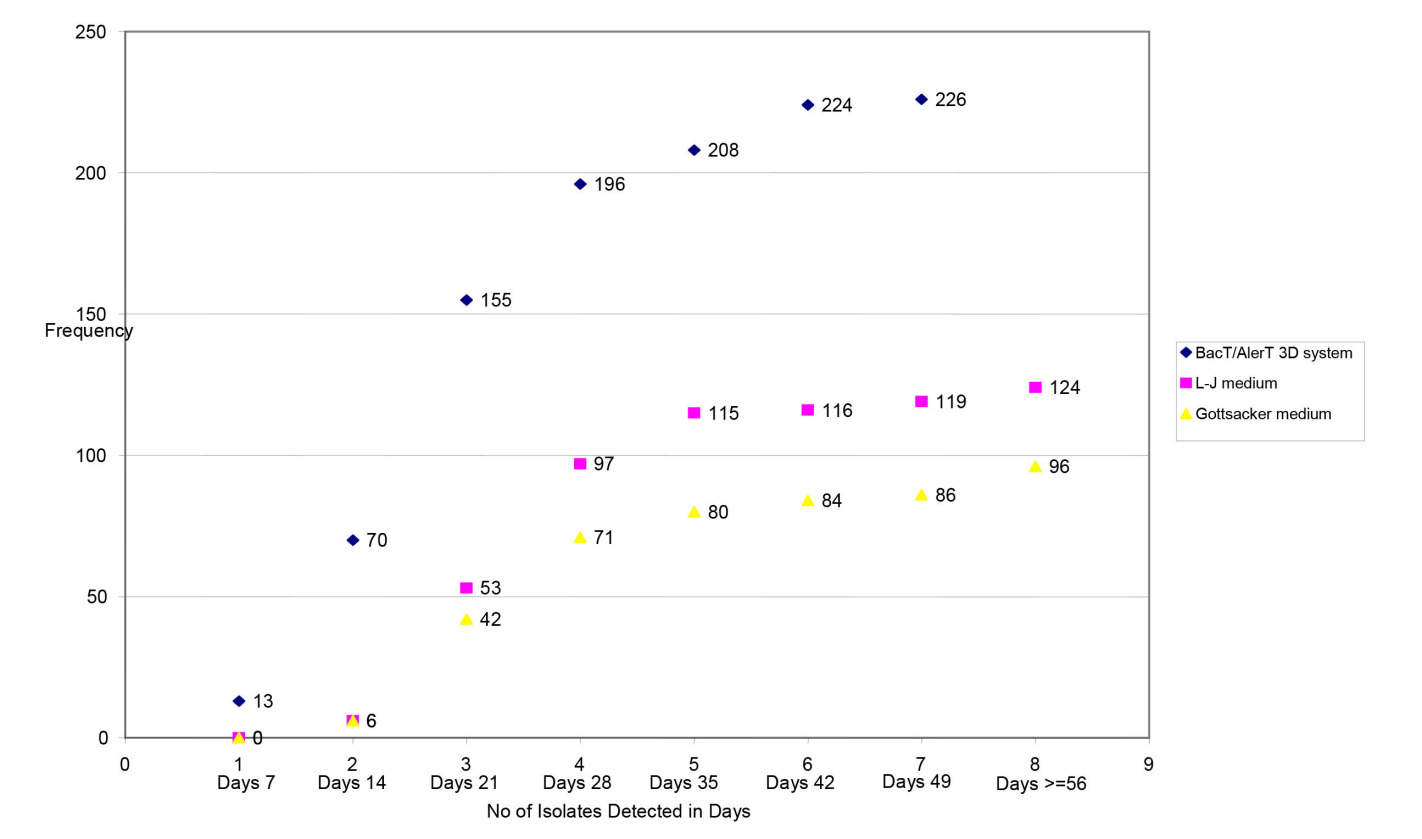
Dot line graph that shows the number of isolates using different culture media at different periods of time, northern Ethiopia.

BacT/AlerT 3D system was found to have high recovery rate 51.7% (n=226) compared to L-J 28.4 % (n=124), Gottsacker 21.9% (n=96) and L-J plus Gottsacker culture methods 30.9% (n=135) (P<0.0001) (Table 2). The median time required for the detection of *M.tuberculosis* complex by the BacT/AlerT 3D system is shorter (median= 20 days, range= 6 to 44 days) than L-J (median= 28 days, range= 17 to 71 days) and Gottsacker (median= 28 days, range= 17 to 73 days) culture methods. BacT/AlerT 3D system was more rapid in the recovery of 5.8% (n=13). However, none of the positive cultures were recovered by L-J and Gottsacker media in the first week of incubation period (Figure 1). 

**Table 2:** Detection of *M. tuberculosis* complex from lymph node aspirates using culture methods and Xpert MTB/RIF assay, northern Ethiopia.

No. with indicated results by								
	Conventional methods*					**Xpert MTB/RIF assay**		
Result	**B N (%)**	**L-J N (%)**	**G N (%)**	**L-J +G N (%)**	**L-J +G+B N(%)**	**Pos N (%)**	**NegN(%)**	**InvN(%)**
**Positive**	226(51.7)	124(28.4)	96(21.9)	135(30.9)**^*a*^**	226(51.7)**^*a*^**	-	-	-
**Negative**	200(45.8)	310(70.9)	337(77.1)	298(68.2)**^*b*^**	200(45.8)**^*b*^**	61(30.5)	132(66.0)	7(3.5)
**Contamination**	11(2.5)	3(0.6)	4(0.9)	4(0.9)**^*c*^**	11(2.5)**^*c*^**	-	-	-
**Total**	437	437	437	437	437	61	132	7

^*^Significant differences (P< 0.0001): BacT/Alert3D vs. L-J medium, BacT/Alert3D vs. Gottsacker medium , BacT/Alert 3D vs. L-J plus Gottsacker media, L-J medium vs. Gottsacker medium, L-J vs. L-J plus Gottsacker media, Gottsacker medium vs. L-J plus Gottsacker media, ^a^ at least one positive, ^b^ all negative, ^c^ at least one contaminated, B, BacT/AlerT 3D system , L-J, Lowenstein Jensen, G, Gottsacker, Pos, positive, Neg, negative, Inv,invalid, N= number,

Most of the patients complained about swelling in the head and neck region with fever, weight loss and/or night sweating. Cough as signs and symptoms was observed in (0.4 %) of the study subjects. Swelling without systemic symptom was observed in 4% of the participants. Presenting systemic symptoms of the study subjects are shown in Table 3. As shown in Table 4, maximum smear 90.9% (n=10) and culture positivity for *M.tuberculosis* 75.7% (n=171) were found to be in the cervical region. Out of the total culture positive cases 14.6% (n=33) were seen in the axillary lymph node group. The associations between infected sites with smear and culture positivity was not statistically significant. On the basis of the hybridization patterns, all the positive culture isolates were identified as *M.tuberculosis*. On the other hand, no *M.bovis* was isolated from any study subjects. 

**Table 3:** Presenting systemic symptoms in 226 patients with tuberculous lymphadenitis.

Clinical presentation	Observation
**Swelling only**	**4.0%**
**Swelling +F**	**0.4%**
**Swelling +C**	**0.4%**
**Swelling +W**	**3.1%**
**Swelling +F+C**	**0.4%**
**Swelling +W+C**	**1.3%**
**Swelling +F+W**	**5.8%**
**Swelling +F+N**	**4.9%**
**Swelling +W+N**	**3.5%**
**Swelling +N+C**	**2.7%**
**Swelling +F+N+C**	**2.2%**
**Swelling +F+W+N**	**47.8%**
**Swelling +W+N+C**	**2.7%**
**Swelling +F+W+N+C**	**20.8%**

F, Fever, C, Cough, W, Weight loss, N, Night sweating

**Table 4:** Lymph node involvement and identification of M.tb complex species by DNA hybridization technology in FNAs from culture positive (n=226) TBLN patients.

Infected sites	Total cases (n=437)	Smear positive (n=11)	P value*	Culture positive (n=226)	P value∆
**Cervical**	321	10 (90.9)	0.302	171(75.7)	0.601
**Axilla**	64	1 (9.1)	1.000	33(14.6)	0.988
**Inguinal**	35	0(0)	1.000	15(6.6)	0.631
**Other**	17	0(0)	1.000	7(3.1)	0.765

^*^ Fisher’s exact test,∆ Chi-square test

The risk factors associated with the occurrence of TBLN are summarized in Table 5. Those pediatric patients (≤ 14 years old) had 2.8 fold higher risks than adult patients (>14 years old). The risk was also two times higher among patients with chronic coughers than those without. However, there was no risk observed between sex, residence, number of persons per room in a house, consumption of uncooked milk, fever, weight loss and night sweating with and without evidence of contact history with TB patients.

**Table 5:** Risk factors associated with the occurrence of TBLN, northern Ethiopia.

Incidence of TBLN in the cases who has contact with TB patients					
Variables	**Yes N** (**%**)	**No N** (**%**)	**Crude OR(95%CI)**	**Adjusted OR(95%CI)**	**P-value**
**Sex**					0.492
**Male**	49(51)	47(49)	1.216(0.717-2.063)	1.230(0.682 -2.218)	
**Female**	60(46.2)	70(53.8)	1.00	1.00	
**Age in years**					0.032
**≤14**	17(65.4)	9(34.6)	2.217(0.944-5.211)	2.774(1.091-7.050)	
**>14**	92(46)	108(54)	1.00	1.00	
**Patients residence**					0.615
**Urban**	30(45.5)	36(54.5)	0.854(0.481-1.519)	0.851(.455-1.593)	
**Rural**	79(49.4)	81(50.6)	1.00	1.00	
No.of persons/room*					0.253
**≤5persons/room**	77(45.8)	91(54.2)	0.687(0.337-1.253)	0.685(.359-1.309)	
**>5persons/room**	32(55.2)	26(44.8)	1.00	1.00	
**Drinking raw milk**					0.528
**Yes**	28(45.2)	34(54.8)	0.834(0.464-1.499)	0.801(0.401-1.598)	
**No**	81(49.7)	82(50.3)	1.00	1.00	
**Fever**					0.628
**Yes**	92(48.4)	98(51.6)	1.049(0.514-2.142)	0.804(0.332-1.945)	
**No**	17(47.2)	19(52.8)	1.00	1.00	
**Weight Loss**					0.188
**Yes**	98(50.5)	96(49.5)	1.494(0.892-4.259)	1.827(0.745-.479)	
**No**	11(34.4)	21(65.6)	1.00	1.00	
**Night sweating**					0.222
**Yes**	99(50.5)	97(49.5)	1.745(0.789-3.860)	1.807(0.700- 4.669)	
**No**	11(36.7)	19(63.3)	1.00	1.00	
**Chronic cough**					0.025
**Yes**	43(59.7)	29(40.3)	1.977(1.119-3.492)	2.017(1.094-3.722)	
**No**	66(42.9)	88(57.1)	1.00	1.00	

^*^ average= 5 persons/ room in a house

## Discussion

TBLN is a common clinical problem and is difficult to diagnose in most rural health centers in Ethiopia, because these health facilities do not offer culture and pathology services. Thus incidence and prevalence of TBLN in the country are poorly documented. The study also revealed that the most affected age group was that in the range of 25-34 years. A female preponderance has also been seen in our study (female: male ratio 1.4:0.7), a finding similar to other authors [14,18,19]. 

Out of the patients described here, the cervical lymph nodes were the prominent sites involved. In this study 76.5% of participants (n=173) have been with matted nodes most often in the cervical regions 57.1% (n=129), which indicates the chronic nature of tuberculosis infection due to late arrival of patients in seeking health care services [4]. Patients with TBLN may have had earlier consultations with traditional healers and come late to health institutions [20]. Moreover, misunderstanding about the causative agent of TBLN could affect the patients’ outlook towards seeking health care services and contribute to the spread of the disease in the community [1]. The health extension workers should be trained to screen TBLN suspects in the community and to link them to the health institutions as early as possible. In addition, health officers and nurses need to be trained on how to collect FNA samples at least from peripheral lymph nodes for further cytological analysis within small health facilities.

In this study, the AFB positive rate of 2.5% falls in between the reported range of 0-32% [7,18,21,22]. The lower sensitivity in the present study was probably due to the small volume of aspirate remaining after culture and smear for cytological assay. In this study, smear positivity was more predictive of a positive culture result (P< 0.0001) in all culture methods agrees with the findings of another study in Ethiopia [7]. This study showed that the prevalence of culture positive TBLN was (51.7%). A considerable proportion (30.5% of 200 cases) of Xpert MTB/RIF test positive cases was culture negative, indicating that Xpert MTB/RIF test increased the rate of detection of mycobacteria in culture negative TBLN cases. When positive result in culture and Xpert MTB/RIF test was considered diagnostic in this study, the overall prevalence of TBLN cases in the study sites were (65.7%). This suggests that cytological examination over diagnosed TBLN by 34.3%. This has implications for poor patient care, improper use of anti-TB drugs and development of drug resistance. The prevalence of TBLN reported in this study is at a considerable deviance from previous reports in the country [6–8,21]. Furthermore, this is in contrast to the national estimate of the prevalence of EPTB patients (34%) [23]. Studies conducted elsewhere in the world reported prevalence of TBLN ranging from 8-55.5% [19,24-27]. The design of the studies, including factors such as sample size and study subjects, the volume of residual FNAs, geographical differences, hospital organization, and the use of different diagnostic methods may have contributed for the discrepancies of the results.

In this study, a rifampicin resistant, which is a surrogate marker for MDR strains, was detected from smear and culture negative cases.This signals an urgent need for definitive diagnosis and treatment of TBLN for effective prevention and control of drug resistant TB in the country. In a resource poor country like Ethiopia, PCR is not performed in most of the cases. However, it should be done at least in ambiguous cases where clinical and cytological cases suggestive of TBLN and AFB stain and culture are negative. Presence of nonviable and scanty number of organisms in lymph node lesion might have accounted for the negative cultures [28]. Previous anti TB drug treatment could also have inhibited the growth of bacilli on culture media. 

Our observation of 437 aspirates showed that BacT/AlerT 3D system provided higher recovery rate than L-J and/or Gottsacker with statistically significant difference (P<0.0001). However, studies elsewhere reported a much higher recovery rate in contrast to our findings [29-33]. This lower recovery rate in our study might be attributed to either the small volume of aspirate remaining after smearing for cytological assay or the presence of dead bacilli during sample storage until processing that failed to grow on the culture. Our findings are in agreement with data from another study [29] inwhich BacT/AlerT 3D system had a short time to detect a positive culture than L-J and/or Gottsacker medium. Likewise, the number of isolates detected from 7 days to 28 days was 86.7%, 78.2% and 73.9% with the BacT/AlerT 3D system, L-J and Gottsacker medium, respectively. In this study, we increased the recommended incubation time 8 weeks to 12 weeks. Our observation suggests that the incubation period was not enough to get an optimal yield with these culture methods, since continued incubation beyond 8 weeks resulted in an increased detection of 15 more isolates. 

For the identification of isolates in the present study, we used GenoType MTBC assay specific for *M.tuberculosis* complex. In our study, *M.tuberculosis* was diagnosed as a causative agent of TBLN. These finding agrees with findings from other studies which also suggested a predominance of *M.tuberculosis* in TBLN in Ethiopia [6,7,20], while bovine transmission would be expected where uncooked milk is consumed. In our study, however, the history of drinking uncooked milk did not show any association with the occurrence of TBLN due to bovine tuberculosis in the study areas. Findings emerge right through the result suggest that *M.bovis* appears to be a rare etiological agent of TBLN in northern Ethiopia. Apart from this study, a study conducted in the country reported *M.bovis* from TBLN cases [5,8,34]. These results were based on biochemical properties which are less reliable and biased to identify the most common *Mycobacterium* species from TBLN cases and need further confirmation with a more sensitive method using PCR. 

Analysis of the risk factors associated with the development of TBLN in the study population indicated that age and cough were the two major predictors of TBLN, which are serious public health concerns that need to be addressed urgently. Results in this study showed pediatric patients (≤ 14 years old) are at high risk to develop TBLN compared to other age groups. This might be due to close intimacy of children with their parents/guardians in home and friends in school. According to our data a person with evidence of chronic cough was two times more likely to develop TBLN. This is in agreement with the previous study conducted in southwest Ethiopia [20]. This indicates the possible route of transmission within the community. However, further investigations might be required to understand the transmission dynamics. In addition, this might point out that the means of acquiring of TBLN is more likely similar to that of pulmonary tuberculosis and to a certain extent explaining why *M.bovis* was not detected in this study. Studies have reported that females were more likely to be positive for TBLN [35]. In this study, however, the rate of TBLN was not significantly different between the males and females, although the majority of females were culture positive for TBLN. Similarly in support of this study, TBLN is reported to be more prevalent in females than males [36].

In conclusion, the study has clearly brought to light that TBLN is highly prevalent in the study sites and that pediatric age and cough are key predictors of the disease. All our isolates were identified as *M. tuberculosis*, indicating that *M.tuberculosis* is still the most common causes of TBLN in Ethiopia. TBLN is an important public health problem that needs to be addressed in the area. It is important to note that MDR strains of TB could be involved and aetiological confirmation and drug sensitivity testing of TBLN isolates should be expanded. Further studies on the *M.tuberculosis* lineages, circulating strains and transmission dynamics, are recommended. 
